# Synergistic Effects of Bortezomib-OV Therapy and Anti-Invasive Strategies in Glioblastoma: A Mathematical Model

**DOI:** 10.3390/cancers11020215

**Published:** 2019-02-13

**Authors:** Yangjin Kim, Junho Lee, Donggu Lee, Hans G. Othmer

**Affiliations:** 1 Department of Mathematics, Konkuk University, Seoul 05029, Korea; ahyouhappy@gmail.com (Y.K.); juneho2222@gmail.com (J.L.); donggu9211@gmail.com (D.L.); 2 School of Mathematics, University of Minnesota, Minneapolis, MN 55455, USA

**Keywords:** glioblastoma, oncolytic virus, mathematical model, bortezomib, ER stress, apoptosis, CSPG

## Abstract

It is well-known that the tumor microenvironment (TME) plays an important role in the regulation of tumor growth and the efficacy of anti-tumor therapies. Recent studies have demonstrated the potential of combination therapies, using oncolytic viruses (OVs) in conjunction with proteosome inhibitors for the treatment of glioblastoma, but the role of the TME in such therapies has not been studied. In this paper, we develop a mathematical model for combination therapies based on the proteosome inhibitor bortezomib and the oncolytic herpes simplex virus (oHSV), with the goal of understanding their roles in bortezomib-induced endoplasmic reticulum (ER) stress, and how the balance between apoptosis and necroptosis is affected by the treatment protocol. We show that the TME plays a significant role in anti-tumor efficacy in OV combination therapy, and illustrate the effect of different spatial patterns of OV injection. The results illustrate a possible phenotypic switch within tumor populations in a given microenvironment, and suggest new anti-invasion therapies.

## 1. Introduction

Glioblastoma multiforme (GBM) is one of the deadliest human cancers, with mean survival times of <15 month because of its aggressive growth and rapid, widespread invasion of the brain [1]. Because it spreads rapidly, a significant number of cancer deaths following resection of a primary tumor are due to secondary tumors that stem from tumor cells that have migrated to other parts of the brain [1,2]. Thus there are two major problems involved; (i) the detection and local treatment of a primary tumor via resection, chemo/radiotherapy, or other means, and (ii) coping with the diffuse, dispersed tumor cells. To date, most therapies focus on the first problem, which is itself complex because of unintended effects of therapies [3], but to increase the mean survival time significantly, the second problem must be confronted.

**Figure 1:**
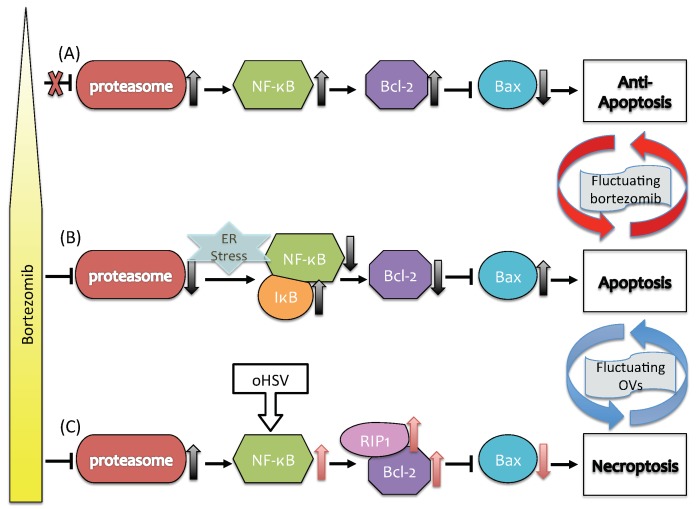
A schematic, non-mechanistic, diagram of the role of bortezomib (BTZ) and onocolytic viruses (OVs) in the regulation of cell death pathways and the effects of fluctuating BTZ and OVs [20,21]. OV activities determine cell apoptosis or necroptosis in response to BTZ (yellow triangle-bar on the left). (**A**) A low BTZ level promotes proteasome activities and leads to upregulation of NFκB/Bcl-2 and downregulation of Bax, thereby suppressing apoptosis. (**B**) A high BTZ level in the absence of OVs suppresses proteasome activities by activating IκB, which in turn inhibits the NFκB/Bcl-2 complex and up-regulates Bax, leading to apoptosis. (**C**) In the presence of OVs, the BTZ-induced endoplasmic reticulum (ER) stress pathway is modified: the NFκB/Bcl-2 complex is upregulated and the Bax level is downregulated. This also results in upregulation of RIP1, leading to necroptosis. Red arrows on the right indicate the switching behavior between an anti-apoptosis mode in (**A**) and the apoptosis state in (**B**) in response to fluctuating BTZ. Blue arrows on the right indicate the switching behavior between a apoptosis mode in (**B**) and the necroptosis state in (**C**) in response to fluctuating OVs.

Since cancer cells are usually highly stressed due to their rapid growth or conditions in the tumor microenvironment (TME), they may contain an excess of un- or misfolded proteins in the endoplasmic reticulum (ER), which leads to ER stress. NF-κB, a regulator of genes that control cell proliferation, is usually sequestered in the cytoplasm and maintained in an inactive state by its inhibitor, IκB. Numerous stimuli, including ER stress, enhance the degradation of IκB, thereby activating NFκB. Upon activation NFκB translocates to the nucleus and activates a wide variety of genes, including *Bcl-2*, which in turn down-regulates the apoptosis gene *Bax* (cf. Figure 1A) [4,5,6,7,8]. In normal tissue this leads to production of IκB, and the feedback loop maintains homeostasis in the tissue [9]. However, a constitutively active NF-κB pathway fosters resistance to anti-cancer therapy [10,11,12] in cancer cells [13,14] and in tumor-associated macrophages [15]. Drugs that activate *Bax* show promise as anti-tumor treatments by inducing apoptosis [5,16]. Necroptosis is a caspase-independent, massive cell death program mediated by high expression levels of reactive oxygen species (ROS) induced by activity of RIP1, a receptor-interacting-protein kinase [17]. Apart from upregulation of *Bcl2L12*, low levels of caspase-8 and upregulated RIP1 were associated with necroptosis in glioma cells [18]. RIP1, a serine/threonine kinase, contains a key death domain and is a major activator of necroptosis, but is not required for activation of NFκB and the apoptotic signaling pathway [17,19].

Proteasomes are protein complexes that regulate degradation and recycling of proteins that control cellular functions such as signal transduction, differentiation and cell death, and their Inhibition leads to the accumulation of various regulatory proteins, including the pro-apoptotic protein p53, in the ER [7]. Thus one therapeutic approach is to use agents such as bortezomib (BTZ), a peptide-based inhibitor of proteosomes, to interfere with protein turnover, and thereby to stimulate immunogenic apoptosis (cf. Figure 1B) [20,21]. BTZ induces apoptosis by inhibiting the phosphorylation of IκB protein, thus inhibiting NF-κB activity [22,23]. While it is used as a single agent or in combination with other radio-/chemo-agents for many cancers, including multiple myeloma, ovarian cancer, and head and neck cancers, different combination strategies are being developed for better efficacy [24,25,26,27] in these and other tumor types.

One promising combination therapy for GBM utilizes an oncolytic virus (OV), specifically, the herpes simplex virus HSV-1, together with BTZ. OV therapy utilizes viruses that are genetically engineered to efficiently target, infect and replicate in cancer cells, with minimal damage to normal cells in surrounding tissue. In the combination BTZ-oHSV approach, HSV-1 exploits the host proteasome [28,29], leading to modified signaling pathways. When cells are infected by viruses, RIP1 may form a complex with RIP3 that mediates necroptosis [30], but RIP1 may have pro-tumor effects as well under different treatment protocols [31,32]. BTZ also increases the expression of HSP60 and HSP90, which leads to expression of receptors that activate natural killer cells, and by promoting breakdown of the cell membrane, necroptosis can induce local changes in the TME that enhance the anti-tumor activity of natural killer cells [33]. In recent experimental studies, Yoo et al. [20,21] demonstrated that the induction of the unfolded protein response (UPR) following BTZ treatment not only improved oHSV replication, but synergistically increased the cancer cell death rate in vitro and in vivo through necroptosis (cf. Figure 1C). They also found that RIP1 and Jun N-terminal kinase (JNK) levels were up-regulated in synergistic cell death of OV-infected cells after BTZ treatment [21]. These findings demonstrated that the synergistic interaction between oHSV and BTZ increases overall therapeutic efficacy. OV therapy is currently being evaluated for its efficacy and safety in many pre-clinical and clinical trials for various cancers [34,35] including gliomas [36,37,38,39,40,41]. While these trials have shown promising results [42], the overall survival data have to be evaluated, and development of efficient, optimized OV treatment strategies remains to be done.

While the BTZ-oHSV combination shows significant positive synergistic effects for local treatment of GBM and leads to localized changes in the TME, it does not address the problem of eliminating the dispersed glioma cells. Chondroitin sulfate proteoglycans (CSPGs) are a major component of the Extracellular matrix (ECM) in brain tissue, and play an important role in regulation of glioma invasion and in the spread of OVs [43]. On the one hand, CSPGs block movement of OVs throughout the tumor and reduce their effectiveness, but on the other hand, other ECM components such as chondroitin sulfate-glycosaminoglycans (CS-GAGs) are reported to block glioma invasion by forming strong adhesion between glioma cells and ECM components [44,45,46]. Kim et al. [47] recently developed a mathematical model of oncolytic virus spread with Chondroitinase ATP-binding cassette (Chase-ABC), a bacterial enzyme that can remove CS-GAGs. This and other work shows that the TME plays a complex role in tumor treatment, and treatment for one effect may enhance other effects.

The model developed herein addresses the two issues mentioned earlier—the local treatment of a tumor, and ways of mitigating the effect of rapid tumor cell dispersal throughout the brain. To address the first issue we define a system of differential equations that describe the evolution of the intracellular variables shown in Figure 1 under specified protocols of BTZ and oHSV treatment. This local component involves the concentration of IκB, NFκB, Bax, and RIP1, and we use this to study the regulation of anti-apoptosis, apoptosis, and necroptosis in response to injections of OVs and BTZ, as well as the anti-tumor efficacy of a BTZ-OV combination therapy, at the tumor level. We then incorporate the local model into a multi-scale model to describe the spatial distribution of uninfected, infected, and dead cancer cells, the density of virus particles, and the concentration of BTZ, This multi-scale model is used to investigate the role of a heterogeneous tumor microenvironment in the regulation of the combination therapy. In silico experiments were performed to investigate the effect of CSPGs on control of invasive tumor cells during the combination therapy, and possible anti-invasion strategies in the complex tumor microenvironment.

## 2. Materials and Methods

### 2.1. The Intracellular Network

**Figure 2:**
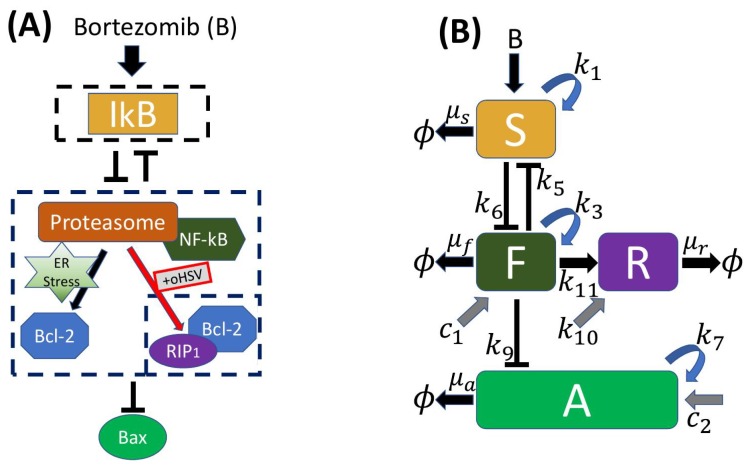
(**A**) A simplified modular model of the network of the IκB-NFκB-Bcl2-Bax-RIP system for anti-apoptosis, apoptosis, and necroptosis [8,20,21,50,51]. The network encodes the mutual antagonism between IκB (top box) and the NFκB-Bcl2 complex (center box), and inhibition of bax by the NFκB-Bcl2 complex. Up- and down-regulation of these modules affects the pro- and anti-apoptosis signaling pathways in response to low and high bortezomib levels *B*. Introduction of oncolytic herpes simplex virus (oHSVs) (small box in the lower right of the center panel) leads to changes in the expression levels of these modules, enhancing the necroptotic pathway. (**B**) A schematic of the interactions amongst the primary variables.

Anti-apoptosis, apoptosis, and necroptosis of tumor cels are mediated by a complex intracellular signaling network of NFκB, its inhibitor Iκ, proteasomes, Bcl-2, and Bax, amongst others, and at present the detailed interactions amongst these components are not known. Therefore we focused on a minimal network of four components shown in Figure 2B that incorporated the primary known interactions, and we described these interactions phenomenologically rather than mechanistically. The best-characterized interactions of the components are between IκB and NFκB. As stated earlier, IκB inhibits NFκB, but it has also been shown that the homolog of NFκB not only activates, but also inhibits the homolog of IκB via the action of a micro-RNA [48,49]. The levels of BTZ and the presence or absence of OVs were treated parametrically in the model for the local dynamics, but the production and diffusion of BTZ were incorporated in the spatially-distributed model later.

The four primary variables in the local model are IκB, NFκB, Bax, and RIP1, which are denoted as S,F,A and *R*. The governing equations of S,F,A and *R* are

(1) $$\begin{array}{c} \frac{dS}{dt}=k_{SB}\frac{B}{k_{12}+k_{13}\left[oHSV\right]}+\frac{k_{1}k_{2}^{2}}{k_{2}^{2}+k_{5}F^{2}}-\mu_{s}S, \end{array}$$

(2) $$\begin{array}{c} \frac{dF}{dt}=c_{1}+\frac{k_{3}k_{4}^{2}}{k_{4}^{2}+k_{6}S^{2}}-\mu_{f}F, \end{array}$$

(3) $$\begin{array}{c} \frac{dA}{dt}=c_{2}+\frac{k_{7}k_{8}^{2}}{k_{8}^{2}+k_{9}F^{2}}-\mu_{a}A. \end{array}$$

(4) $$\begin{array}{c} \frac{dR}{dt}=k_{10}+k_{11}\left[oHSV\right]F-\mu_{r}R. \end{array}$$

Here *B* encodes the level of BTZ, which serves as a surrogate for the signaling pathways from BTZ to IκB, [oHSV] is a two-state switch for the oncolytic virus, with [oHSV] = $\frac{v}{k+v}$ where *v* is the OV density, as introduced below, and *k* is the Hill type parameter, giving [oHSV] = 0 (1) below (above) a threshold of virus. When B=0 the first two equations reflect the fact that IκB and NFκB repress one another. In the first equation $k_{2}$ and $k_{5}$ denote the coefficients in the Hill function that models the inhibition—here assumed to be quadratic—while $k_{1}$ encodes the strength of this inhibition on IκB. Similarly, $c_{1},c_{2},k_{10}$ encode inputs to the NFκB-Bcl-2 complex, Bax, and RIP1, pathways, resp., $k_{3}$ and $k_{7}$ are the autocatalytic enhancement parameters for NFκB-Bcl-2 complex and Bax, resp., $k_{4}$ and $k_{6}$ ($k_{8}$ and $k_{9}$) denote the coefficients in the Hill function that models inhibition of the NFκB-Bcl-2 complex, (Bax), resp. Finally, $\mu_{s},\;\mu_{f},\;\mu_{a}$ and $\mu_{r}$ represent the decay rates of the species.

It should be noted that the equations for IκB and NFκB are decoupled from the equations for Bax and RIP1. Since the interaction between *S* and *F* is mutual repression, the parameters could easily be chosen to produce a region of bistability, which defines a switch, for a suitable range of parameters in the Hill functions. However, as we show later, the parameters used here do not produce a switch, but rather a rapid transition from a high to low value of NFκB as B is varied.

The third equation describes the evolution of Bax, which in our formulation is inhibited by NFκB via the second term in Equation (3). This again is a simplification of the network from NFκB to Bax, as suggested in Figure 1A, but reflects the fact that NFκB↑ leads to Bax↓. The last equation governs RIP1, and reflects the fact that RIP1 is upregulated in response to combination treatment with BTZ and oHSV, leading to necroptotic cell death [21]. In the absence of treatment, glioma cells maintain upregulated Bcl-2 and develop resistance to cytotoxic and pro-apoptotic agents [17,52]. BTZ induces apoptosis via ER stress in the absence of oHSV but, in the presence of OVs, the combination OVs+BTZ induces increased ROS and JNK activity, which leads to the critical transition from either apoptotic or anti-apoptotic pathways to necroptosis [21]. Therefore, the term $k_{11}\left[oHSV\right]F$ is activated only in the presence of sufficient oHSV, and the strength of the effect depends on the level of NFκB.

The values of all parameters used in these equations are given in Table 1.

### 2.2. The Spatially-Distributed Component of the Model

**Figure 3:**
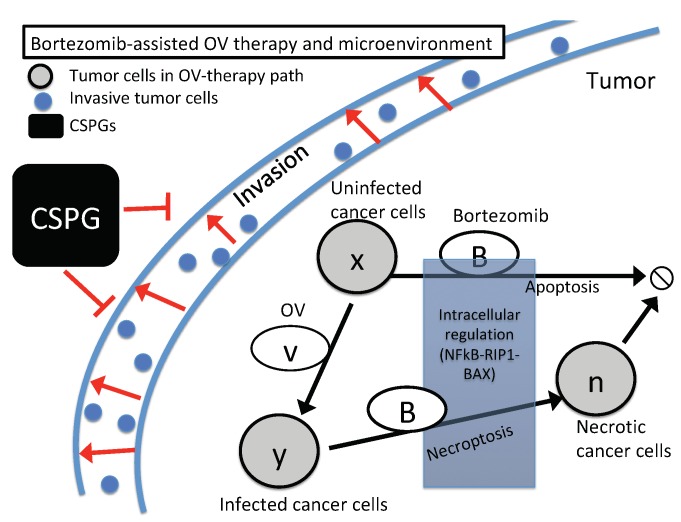
A regulatory network involving OV therapy (gray circles) and the tumor microenvironment (TME) in BTZ-assisted OV therapy. Arrows indicate induction and activation. Hammerheads indicate inhibition.

In the second component of modeling we expanded the local model to describe the evolution of various states of glioma cells and other factors in both space and time. To do this we added partial differential equations that describe the spatial distributions of three states of glioma cells—those that are uninfected by virus, infected, and dead. The densities of these are denoted x,y and *n*, resp., and we also describe the density of virus particles (*v*), and the concentration of BTZ (*B*). The postulated interactions of these components are shown in Figure 3. The spatial distributions are restricted to a planar surface, and thus all variables are functions of time and two spatial variables.

The generic form of the evolution equation for all spatially-distributed components is

(5) $$\frac{\partial w}{\partial t}=-\nabla \cdot J_{w}+P_{w},$$

where *w* is one of x, y, n, v or *B*, $J_{w}$ is the flux of that species, $P_{w}$ is the birth/death (or production/destruction) rate of that species, and ∇ is the divergence operator in two dimensions. We suppose that all components are restricted to a closed bounded domain in the plane and impose the no-flux condition $n\cdot J_{w}=0$ on the boundary, where *n* is normal to the boundary.

We assume that the fluxes of mobile species (all but dead cells *n*) are due to Fickian diffusion, and thus the flux of any species other than *n* is given by

(6) $$J_{w}=-D_{w}\nabla w.$$

The birth/death of the production/destruction rate of each species is the sum of all processes that lead to creation or destruction of that species [20,47,51,58].

For the uninfected cells these processes are proliferation, which we assume follows a logistic growth law, apoptosis, necroptosis, and viral infection. Therefore

(7) $$P_{x}=\lambda x\left(1-x/x_{0}\right)-\beta xv-\beta_{1}xBI_{apop}-\beta_{3}xvI_{Necrop},$$

where λ is the proliferation rate of uninfected glioma cells whose carrying capacity is $x_{0}$, β is the infection rate in the absence of BTZ, $\beta_{1}$ is the BTZ-induced apoptosis of unifected cells, $\beta_{3}$ is the BTZ-induced necroptotic cell death rate in the presence of OVs. Further, $I_{apop}\left(\cdot \right)$ and $I_{necroptosis}\left(\cdot \right)$ are the indicator or characteristic functions of the apoptotic and necrotic regions in F,A,R space. These functions are either one or zero, depending on whether F,A and *R* are in specified ranges defined in the following section. Thus, the governing equation for *x* is

(8) $$\frac{\partial x}{\partial t}=\nabla \cdot \left(D_{1}\nabla x\right)+\lambda x\left(1-x/x_{0}\right)-\beta xv-\beta_{1}xBI_{apop}-\beta_{3}xvI_{Necrop}.$$

We assume that (i) dying cells do not move, (ii) infected tumor cells become dying cells at a rate δ, (iii) dying cells are cleared from the system at a rate μ, and (iv) infected cells diffuse at a rate $D_{2}$. From this it follows that the governing equations for densities of uninfected (*y*) and dead cells (*n*) are as follows:

(9) $$\begin{array}{c} \frac{\partial y}{\partial t}=\nabla \cdot \left(D_{2}\nabla y\right)+\beta xv-\delta y+\beta_{3}xvI_{Necrop} \end{array}$$

(10) $$\begin{array}{c} \frac{\partial n}{\partial t}=\delta y-\mu n. \end{array}$$

The oncolytic virus is replication-competent, and BTZ enhances viral replication by a factor proportional to *B* [20]. We denote by *b* the number of viral particles released after OV-mediated lysis of infected cancer cells. Hence, the equation for *v* is the following:

(11) $$\frac{\partial v}{\partial t}=\nabla \cdot \left(D_{v}\nabla v\right)+b\delta y\left(1+\alpha_{1}B\right)-\gamma v.$$

BTZ is supplied to the glioma via direct injection and diffusion through the brain tissue. We took into account the consumption from the internalization of BTZ in tumor cells and natural decay at rate $\mu_{B}$. Hence, the governing equation is

(12) $$\frac{\partial B}{\partial t}=\nabla \cdot \left(D_{B}\nabla B\right)+I_{B}-\left(\mu_{1}x+\mu_{2}y\right)\frac{B}{k_{B}+B}-\mu_{B}B,$$

where $D_{B}$ is the diffusion coefficient of BTZ, $I_{B}$ is the effective injection rate of BTZ, $\mu_{1},\mu_{2}$ are consumption rate of BTZ by uninfected and infected tumor cells, respectively, and $k_{B}$ is the Hill type coefficient.

The list of parameters and their values are given in Table 2 for the distributed variables. In the foregoing Equations (1)–(12) are stated in terms of dimensional quantities. These are cast into dimensionless form for computational purposes in the Supplementary Information file, and the basis for the parameter estimations is given there. The simulations were done using the alternating direction implicit method and the non-linear solver, nksol, for algebraic systems. The equations were solved on a regular uniform spatial grid ($h_{x}=0.01,\;h_{y}=0.01$) using an adaptive time-stepping method [59].

## 3. Results

### 3.1. Intracellular Dynamics

**Figure 4:**
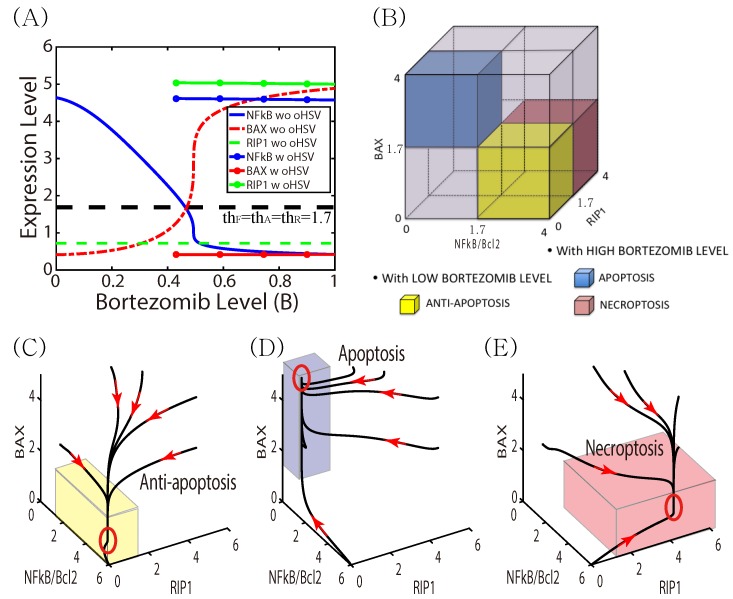
The dependence of the intracellular variables (IκB-NFκB-Bax-RIP1) on BTZ, and characterization of the cell death program (anti-apoptosis, apoptosis, and necroptosis). (**A**) BTZ levels produce an effective on-off switch for control of NFκB, Bax, and RIP1 levels, and activate the cell death program: anti-apoptosis, apoptosis, or necroptosis. (**B**) Characterization of cell death in the NFκB-RIP1-Bax state space, where the labeled domains are defined in Equations (13)–(15). (**C**–**E**) Dynamics of the core control system in response to low (B=0.0 in (**C**)) and high (G=1.0 in (**D**,**E**)) BTZ levels in the absence (**C**,**D**) and presence (**E**) of OVs. The stable steady states in each subframe lie within the red circles.

We recall that low levels of NFκB and Bcl-2 lead to up-regulated Bax and induce apoptosis, while over-expression of NFκB and Bcl-2 leads to down-regulation of Bax and up-regulation of RIP1, which induces necroptosis (Figure 1). As a first step toward understanding the effects of BTZ and oHSV on cell death in the full model, we analyzed how the BTZ level (*B*) affects the levels of the key effectors of cell death (F,A,R) in the intracellular model. When the core IκB-NFκB-Bax-RIP1 system (1)–(4) is at a steady state, we can solve the algebraic equations for the steady-state values of F,A,R as a function of the extracellular BTZ level (*B*), and we denoted the resulting values by $F^{s},A^{s},R^{s}$. Figure 4A shows the graphs of F=F(B) (blue), A=A(B) (red), R=R(B) (green) in the absence (without circle markers) and presence (with circle markers) of OVs. The response curves of NFκB and Bax show that Bax inherits the IκB-NFκB mutual antagonism, with a crossover at B∼0.5. We define thresholds for the variables at the expression level of NFκB at crossover as $th_{F}$ = 1.7 for NFκB, $th_{A}$ = 1.7 for Bax, and $th_{R}$ =1.7 for RIP1, and we use these to define the anti-apoptotic ($T_{t}$), apoptotic ($T_{a}$), and necroptotic ($T_{n}$) regions as

(13) $$\begin{array}{c} T_{t}=\left\{\left(F,A,R\right)\in R^{3}:F>th_{F},\;A<th_{A},\;R<th_{R}\right\}, \end{array}$$

(14) $$\begin{array}{c} T_{a}=\left\{\left(F,A,R\right)\in R^{3}:F<th_{F},\;A>th_{A},\;R<th_{R}\right\}, \end{array}$$

(15) $$\begin{array}{c} T_{n}=\left\{\left(F,A,R\right)\in R^{3}:F>th_{F},\;A<th_{A},\;R>th_{R}\right\}, \end{array}$$

respectively (see Figure 4B).

In the absence of OV therapy, and under low levels of BTZ, the system of Equations (1)–(4) exhibits low Bax levels, high *F* levels, and low *A* and *R* levels, and the cells are in the anti-apoptotic state $T_{t}$. Under these conditions the cancer cells would continue to grow as *B* increases until it reaches the crossover point (∼0.5). In the vicinity of this point the Bax level rises rapidly, which leads to down-regulated NFκB, and the cells are characterized as being in the apoptotic state. For an illustration of the dynamics, Figure 4C,D show the dynamics of the core control system in response to low (B=0.0; Figure 4C) and high (B=1.0; Figure 4D) BTZ levels. Starting from various initial conditions ((S,F,A,R)(0) = (0, 0, 2, 1), (0, 1, 5, 3), (0, 2, 5, 4), (0, 5, 5, 5), (0, 5, 3, 5), (0, 6, 0, 0).) the dynamics lead to either the $T_{t}$, $T_{a}$ or $T_{n}$ state. For a low BTZ level, the system converges to the unique steady state ($(F^{s},A^{s},R^{s}$) (∼(4.64,0.41,0.72)) in the anti-apoptotic region where Bax (*A*) and RIP1 (*R*) expressions are low, but NFκB (*F*) activity is high. On the other hand, for high BTZ levels (B>0.5), there is only one stable steady state ($(F^{s},A^{s},R^{s})∼$(0.42, 4.89, 0.72)) in the apoptotic region $T_{a}$ where NFκB and RIP1 expressions are low but Bax activity is high. However, the presence of oHSV leads to upregulation of RIP1 and NFκB and downregulation of Bax ($(F^{s},A^{s},R^{s}$) (∼(4.57, 0.42, 5.0)) in response to the high BTZ level (Figure 4E). This dichotomous behavior of the NFκB and Bax modules via ER stress in response to high and low BTZ levels are well known experimentally [13,20,21,30,70], but have not been replicated with a mathematical model heretofore.

**Figure 5:**
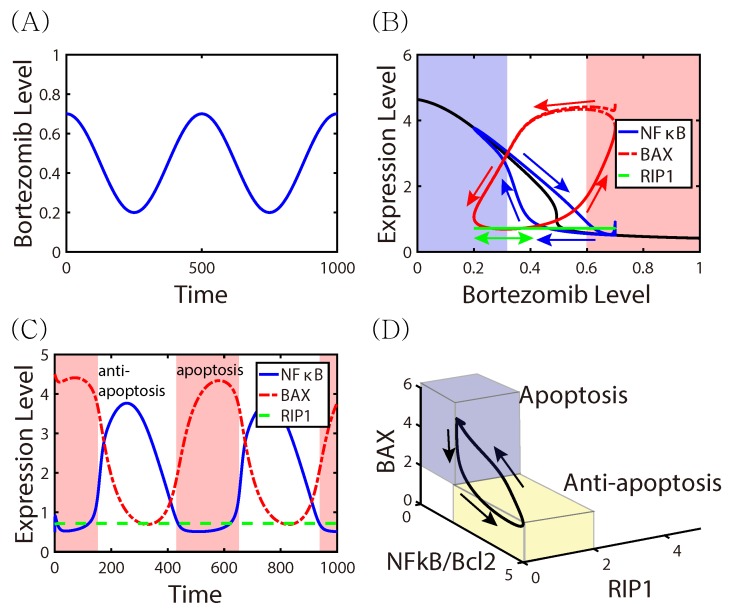
The effect of fluctuating BTZ on the transition between $T_{t}$ and $T_{a}$ in the absence of OVs. (**A**) A time-dependent BTZ level (B(t)=0.25×cos(πt/250)+0.45) was assigned for a periodic injection of BTZ in tumor microenvironment. (**B**) Trajectories of solutions (F(t),B(t)) and (A(t),B(t))) in response to BTZ in (**A**). The black curve represents the upper and lower branches of steady states (F−B bifurcation loop in Figure 4A). Red and blue arrows = solution flow of Bax and NFκB-Bcl2, respectively. Fluctuating BTZ levels induce transitions between anti-apoptotic and apoptotic status. (**C**) Time courses of concentrations of intracellular variables (NFκB-Bcl2 (blue solid), Bax (red dashed), and RIP1 (green dashed)) in response to periodic *B* injection in (**A**). (**D**) Trajectories of solutions corresponding to (**C**) in the F−A−R space. Initial conditions: B(0)=0.7,S(0)=0,F(0)=0.5,A(0)=4.5,R(0)=0.7.

One can predict the qualitative responses of the solution components to time-varying BTZ levels in the absence of OVs from Figure 4, and these are shown explicitly for a periodic variation in Figure 5. The BTZ level is defined as B(t)=0.25×cos(πt/250)+0.45), and starting at high BTZ and Bax, the trajectory of Bax follows the upper branch of the red loop for decreasing BTZ until BTZ drops to ∼0.2, whereupon it follows the increasing part of the cycle. The blue solid curve and blue arrows represent NFκB and its flow, where NFκB decreases along the upper branch as BTZ increases, while it increases along the lower branch as BTZ decreases. Figure 5C shows the solutions (F(t),A(t),R(t)) as a function of their position in the BTZ cycle. Fluctuating BTZ induces the up- or down-regulation of NFκB, leading to periodic transitions between $T_{t}$ (the white region) and $T_{a}$ (the pink region). The corresponding solutions are shown in the F−A−R space (Figure 5D).

### 3.2. Spatial Effects

**Figure 6:**
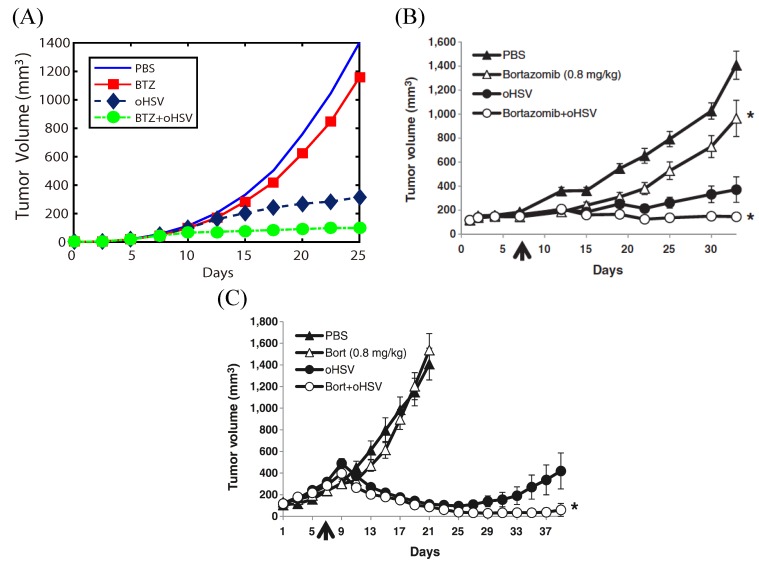
(**A**) Simulation results of tumor volume from simulations for PBS (blue solid), BTZ treatment (red solid square), oHSV treatment (black dotted diamond), and combination treatment (oHSV+BTZ; green dotted diamond). (**B**,**C**) Experimental results on tumor growth in response to the random motility of virus (PBS), BTZ treatment, oHSV treatment and combination therapy oHSVs+BTZ [20]: Athymic nude mice were subcutaneously implanted with CAL27 head and neck cancer cells (**B**) and U251T3 glioma cells (**C**). PBS or BTZ (0.8 mg/kg) treatments were administered through intraperitoneal injection twice a week. Following one week of BTZ treatment, mice were injected intratumorally with HBSS or oHSV ($1\times 10^{5}$ pfu in (**B**) and $5\times 10^{4}$ pfu of 34.5ENVE in (**C**)). Figure 6B,C and legend are taken from [20].

Next, we compared experimental results with simulation results for various treatments, using the full model that incorporates spatial variations of components. Figure 6 shows the time course of the tumor volume for wild type (PBS), BTZ treatment, oHSV treatment, and BTZ+oHSV treatment with basic parameter values of BTZ injection (Tumor volumes are calculated based on the calculated tumor diameter (D=2r, *r* = radius, volume (*V*)$=\left(4\pi /3\right)\times {\left(D/2\right)}^{3}$) in simulation (Figure 6A) [47] and on tumor length (*L*) and width (*W*) using the formula $V=0.5\;LW^{2}$ for subcutaneous studies in the experiments (Figure 6B,C) [20]). One sees that when BTZ is combined with oHSV therapy, the tumor size is significantly reduced compared with other treatment protocols [20]. Of course, the killing rate of a tumor in the presence of BTZ may depend on various cell lines and tumor microenvironments in experiments. For example, mice implanted with CAL27 head and neck cancer cells (Figure 6B) and U251T3 glioma cells (Figure 6C) show slightly different time curves of tumor growth [20]. However, the overall anti-tumor efficacy of the combined therapy BTZ+oHSV was evident in these experiments. Results from the mathematical model shown in Figure 6A are in good agreement with experimental data, in particular with the growth pattern of CAL27 head and neck cancer cell lines (Figure 6B) in [20]. Moreover, the overall synergistic effect of bortezomib on tumor growth is similar to that in U251T3 glioma cells (Figure 6C). In general, subcutaneous head and neck tumors and orthotopic glioma models present different growth and invasion patterns, and the location of the tumor might influence the experimental data. In the subcutaneous tumors, the tumor usually does not grow invasive as in the brain, but is surrounded by a capsule. The details of the growth patterns can be fitted to experimental data using different parameters in the mathematical model (cf. Figure S7 in supplementary information Appendix in [51].) The mathematical model developed here presents the general framework of tumor growth in response to the combination therapy OVs+BTZ so that these results can be used to explore the various tumor dynamics in the presence of CSPGs and other microenvironments in brain tissue.

**Figure 7:**
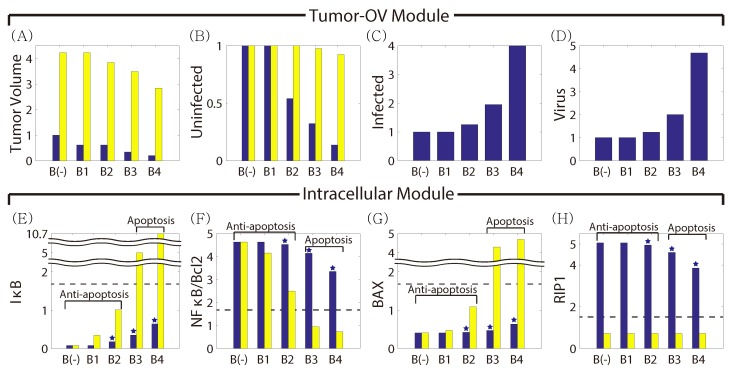
Effect of BTZ on anti-tumor efficacy in the absence (yellow bar) and presence (blue bar) of OVs. (**A**) Normalized tumor volume at day 20 for various BTZ supply rates ($I_{B}=0\left(B\left(-\right)\right),$ $3.3\;\times \;10^{-2}\left(B1\right),7.7\times 10^{-2}\left(B2\right),1.8\times 10^{-1}\left(B3^{*}\right),3.3\;\times \;10^{-1}\left(B4\right)$). (**B**–**D**) Relative populations of uninfected cancer cells (**B**), infected cancer cells (**C**), and oHSV (**D**). (**E**–**H**) levels of intracellular variables, IκB (**E**), NFκB (**F**), Bax (**G**), and RIP1 (**H**).

In Figure 7, we show the effect on cell death of altering the BTZ dosage in the absence and presence of OVs. In the presence of OVs, tumor volume was decreased as the BTZ level was increased compared to the control case (*B*(−); $I_{B}=0.0$), leading to higher anti-tumor efficacy (blue; Figure 7A). BTZ also enhanced virus replication (blue; Figure 7D) and infected cell population (blue; Figure 7C), leading to higher necrosis and higher OV-enhanced cell death. The enhanced OV activities in the presence of BTZ is in good agreement with experimental observations in previous studies [20,21]. However, the anti-tumoral effect of the apoptotic pathways via BTZ alone was minimal. For example, ER stress and apoptosis (signaling pathways in Figure 7E–H) were insignificant, leading to little change in anti-tumor efficacy (yellow, Figure 7A). This relatively low response for BTZ treatment was also observed in the experiments [20,21]. These results confirm the synergistic effect of BTZ and OV treatment on anti-tumor efficacy.

**Figure 8:**
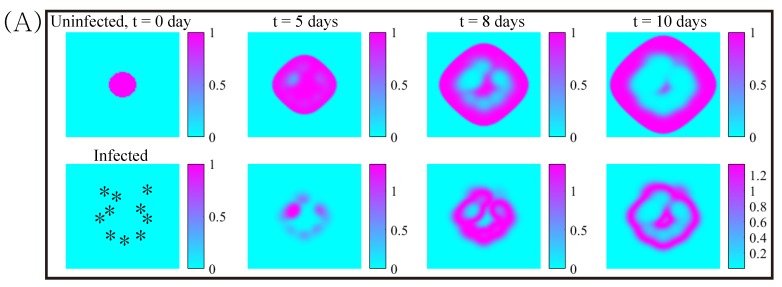

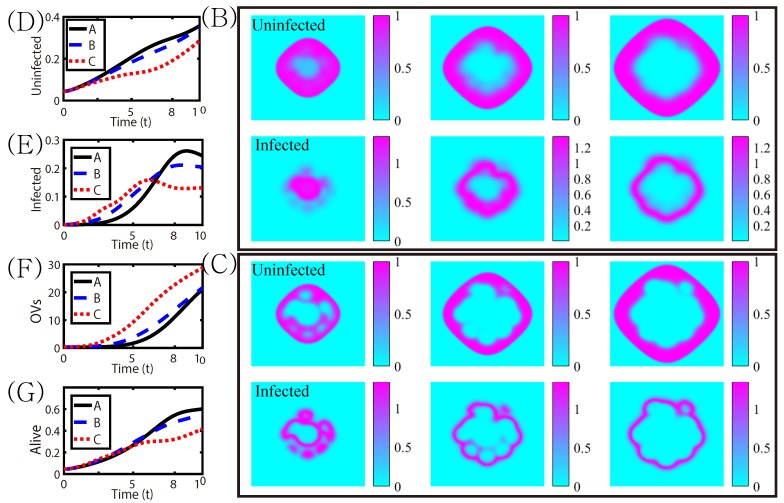
The dynamics of tumor growth and cell death under the combination therapy. (**A**) Profiles of solutions of uninfected (*x*) and infected (*y*) tumor cells at time t=0,5,8,10 days in response to OV injections on the periphery of the growing tumor. (**B**,**C**) Profiles of solutions in response to OV injections on the periphery and center of the tumor with OV only (**B**) and combination therapy OVs+BTZ; (**C**) at the times shown in the header for (**A**); (**D**–**F**) time courses of uninfected tumor populations (**D**), infected tumor population (**E**), and OVs (**F**) in three cases of (**A**–**C**). (**G**) Time courses of the populations of alive (ininfected+infected) tumor cells in three cases of (**A**–**C**).

In Figure 8, we show the effects of three different strategies for killing cancer cells. When a bolus of OVs were injected on the periphery of the growing tumor (asterisks in the lower panel of Figure 8A), the growing tumor cells were infected and killed. However, some of the tumor cells avoided the OV attacks and regrew outside the infected areas (upper panel in Figure 8A). When more OVs were injected in the center of the tumor core in addition to the periphery, tumor cells were infected in the central region of the tumor and more cells die (Figure 8B). When BTZ was added at the same injection points, OVs were amplified (Figure 8F) and more tumor cells were infected (Figure 8E), increasing the anti-tumor efficacy (Figure 8G). However, in all cases, there was a large growing ring of tumor cells that survive treatment. It is known that the location of injection sites of OVs for multifocal glioma [71,72,73] and anti-tumor immunity [74,75,76,77] may influence the anti-tumor efficacy and killing of invasive glioma cells.

**Figure 9:**
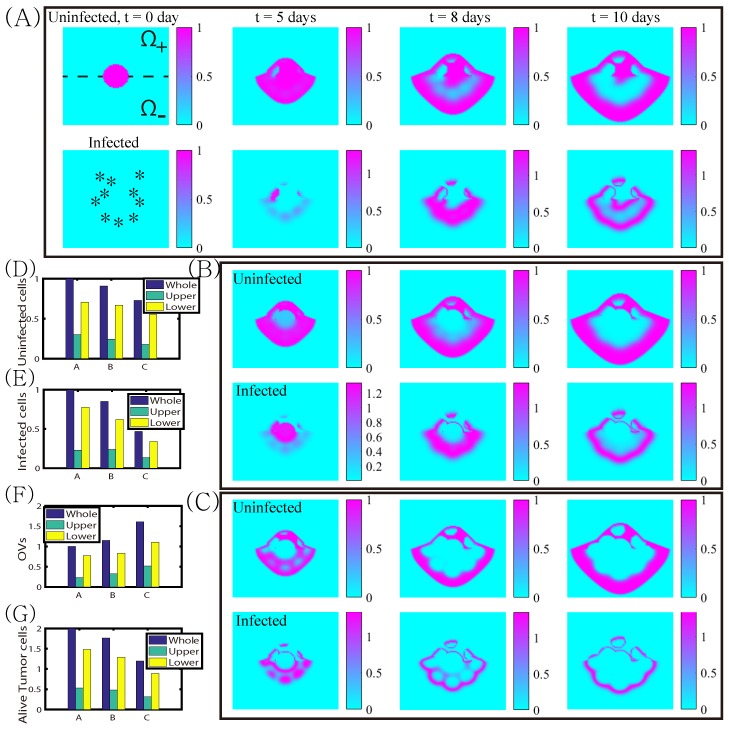
Dynamics of tumor growth and cell killing under combination therapy in different TMEs. In $\Omega_{+}$ the diffusivities of diffusible variables are reduced 100-fold. (**A**) Profiles of solutions of uninfected (*x*) and infected (*y*) tumor cells at time t=0,5,8,10day in response to OV injections on the periphery of the growing tumor. (**B**,**C**) Profiles of solutions in response to OV injections alone (**B**) and combination therapy OVs+BTZ; (**C**) on the periphery and center of the tumor at the corresponding time. (**D**–**G**) Populations of uninfected tumor cells (**D**), infected tumor cells (**E**), OVs (**F**), and alive tumor cells (**G**) at final time. * Whole population (blue; Ω), population in the upper domain (green; $\Omega_{+}$), and population in the lower domain (yellow; $\Omega_{-}$).

Next we investigated how the TME influences the effect of the combination therapy. Figure 9A–C shows the spatial profiles of uninfected and infected tumor densities in response to OV injection on the periphery, OV injection on the periphery and center of the tumor, and the combination therapy OVs+BTZ. In all cases the diffusion coefficients of diffusible variables were 100-fold less in the upper half $\Omega_{+}$ of the computational domain Ω=[0,1]×[0,1] than in the lower half $\Omega_{-}$. The injection sites were marked in asterisks in the lower t = 0 panel in Figure 9A. While the combination therapy resulted in the enhanced anti-tumor efficacy compared to OV therapies, tumor growth (Figure 9G) and OV spread (Figure 9E,F) are slower in the upper domain in all cases. A challenging microenvironment such as gray matter or dense ECM structure in the brain has been shown to prevent tumor invasion [78,79] and limit viral spread in the tissue [43,80]. Therefore, ECM-degrading substances such as Relaxin/decorin [81,82] and Chase-ABC [43] have been suggested to improve the anti-tumor efficacy [83,84,85,86,87,88]. MRI histological analysis showed the presence of preferred migratory paths of glioma cells and different diffuse growth patterns in complex brain microenvironment [89]. This undoubtedly involves chemotaxis of glioma cells, but this has not been included in the present model. Nonetheless, our simulation results illustrate how the tumor microenvironment can affect the OVs+BTZ combination therapy.

### 3.3. Effect of CSPGs on Glioma Invasion and Anti-Invasion Strategies

**Figure 10:**
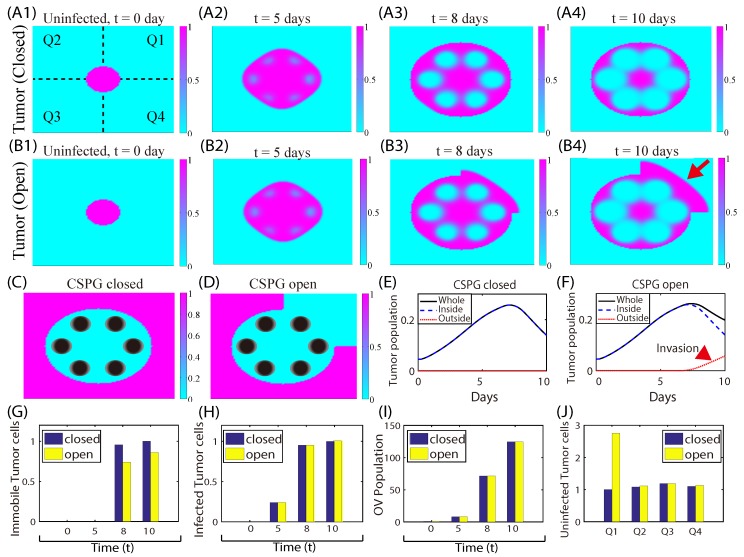
The role of the TME in the regulation of glioma invasion. (**A**) Profiles of uninfected tumor cells (*x*) in response to OVs and BTZ in the presence of CSPG surrounding the tumor at t= 0, 5, 8, 10 days. OVs (*v*) and BTZ (*B*) were injected in the interior of CSPG bands marked in black circles in (**C**,**D**). (**B**) Profiles of uninfected tumor cells (*x*) under the same conditions, but with a partial gap in Q1. One sees that some of surviving tumor cells invade the brain tissue in $Q_{1}$. (**C**,**D**) The spatial distribution of CSPG in the closed (**C**) and open cases (**D**) corresponding to (**A**,**B**), respectively. Spatial locations of injection sites for both OVs and BTZ are marked in black filled circles in the interior. (**E**,**F**) The time courses of the tumor index (the normalized tumor cell populations); total tumor population (black solid), tumor population inside the CSPG ring structure (blue dashed), and invasive tumor cells outside the ring structure (red dotted). (**G**) Immobile tumor cells near the CSPG boundary in closed and open cases at t=0,5,8,10 days. (**H**,**I**) Infected tumor population (**H**) and OV (**I**) population in closed and open cases. (**J**) Uninfected tumor populations in the four quadrants at the final time in the closed (blue) and open (yellow) cases.

CSPGs, which are major parts of the ECM in the brain, can form heterogenous structures such as a ring with partial openings [90] or heterogenous patches [91], and it is known that CSPGs characterize the invasive and non-invasive phenotypes of tumors [44,92]. In the following computations the four quadrants in the computational domain are marked Q1-Q4—see Figure 10A1—and the spatial distributions of CSPGs surrounding the tumor are shown in Figure 10C (closed) and Figure 10D (open). Figure 10A,B show the time courses of uninfected tumor density at t= 0 (A1, B1), 5 (A2, B2), 8 (A3, B3), 10 days (A4, B4) in response to OVs+BTZ injections in the presence of a CSPG ring surrounding the tumor without (Figure 10A), and with (Figure 10B) an open section in the first quadrant. Both OVs and BTZ were injected at the six spots (black disks in Figure 10C) in the interior region. Figure 10E,F show the time courses of tumor populations within the whole area (black solid), inside (blue dashed), and outside (red dotted) the CSPG surrounding for the closed (Figure 10E) and open (Figure 10F) cases, respectively.

In both cases, uninfected tumor cells initially invaded the nearby surrounding tissue after treatment with OVs and BTZ. In the closed case, cell invasion was blocked at the CSPG barrier in all quadrants at around t=8 days. The blockage was incorporated in the model by setting the diffusion coefficient of the cells to zero at the boundary of the magenta-colored region in Figure 10C,D. In the presence of the open sector, some uninfected tumor cells in the Q1 region invade the brain tissue through the open gap in the CSPG barrier (red arrow in Figure 10B4) (see also the increasing population (red arrowhead) of invasive cells in Figure 10F). Figure 10G shows the normalized populations of uninfected tumor cells that are strongly adhered to the CSPG boundary at t=0,5,8,10 days in the open and closed regions. Figure 10H,I show the normalized populations of infected tumor cells and OVs at the corresponding time points for open (yellow) and closed (blue) cases, respectively. No significant differences in OV infection activities are observed between the open and closed cases. The relative amounts of uninfected tumor cells at 20 days in the four quadrants are shown in more detail in the open (yellow) and closed cases (blue) in Figure 10J. In the open case, the total number of cells was higher and more are present in Q1, while the numbers in Q1-Q4 were roughly equal in the closed case. These results show the critical role of the CSPG distribution in the regulation of glioma invasion, even in the presence of OV therapy. This also suggests the possibility of blocking aggressive glioma invasion by rearrangement or manipulation of CSPG ECM through LAR-CSGAG interaction on the periphery of a growing tumor [44,92].

**Figure 11:**
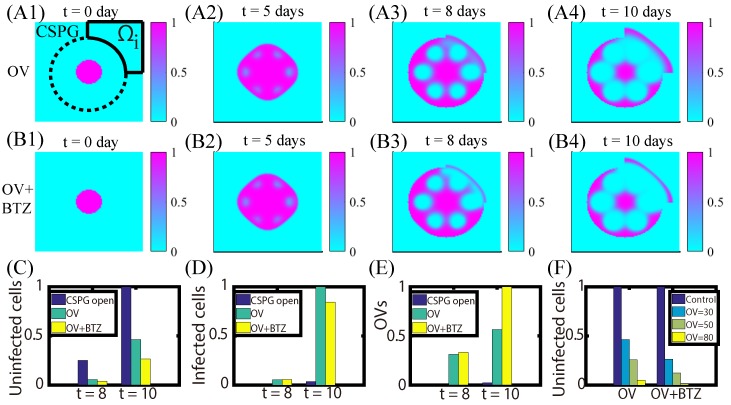
Anti-invasion strategies. (**A**,**B**) The distributions of uninfected tumor cells at time t= 0, 5, 8, 10 days in response to injection of OVs (**A1**–**A4**), or OVs+BTZ (**B1**–**B4**), in the invasion area ($\Omega_{i}$) and OV-BTZ treatment in the interior. (**C**–**E**) Populations of uninfected tumor cells (**C**), infected tumor cells (**D**), and OVs (**E**), respectively, in the invasion region ($\Omega_{i}$) of the Q1 area at t=8,10 days in response to no treatment (blue), OV treatment (green), and combination treatment OVs+BTZ (yellow) in the $\Omega_{i}$ area. (**F**) Populations of invasive tumor cells in $\Omega_{i}$ at final time for control (without OV injection; blue) and various levels of OVs (left column) and OVs+BTZ (right column) on the invasion sites.

In Figure 11 we investigated anti-invasion strategies in the presence of a CSPG band with a partial gap. When OVs were injected in the invasion area ($\Omega_{i}$ in Figure 11A1) in addition to the interior of the CSPG band, some of the surviving invasive cells were killed by these OVs (Figure 11A). However, OVs alone were not enough to completely block the invasion of the glioma cells. When BTZ was added at the same invasive site, the anti-invasion efficacy is increased Figure 11B. Therefore, strategic injection of OVs+BTZ may be effective in controlling the invasive multi-focal gliomas [93]. Figure 11C–E show populations of uninfected tumor cells (C), infected tumor cells (D), and OVs (E), respectively, in the invasion region ($\Omega_{i}$) of Q1 at t=8,10 days. Higher rates of OV replication and infection compared to the control lead to increased killing of invasive tumor cells in the invasive area. Figure 11F shows populations of invasive tumor cells in $\Omega_{i}$ at the final time *t* = 10 days for control (without OV injection; blue) and various levels of OVs (left column) and OVs+BTZ (right column) on the invasion sites.

### 3.4. Anti-Invasion Strategies with OV Therapy: Localization

**Figure 12:**
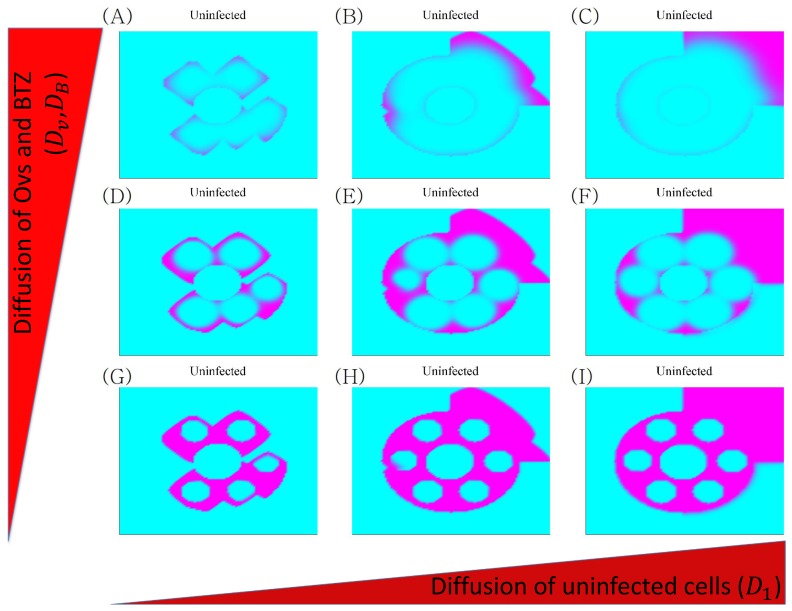
Effect of the spreading speed of OVs and tumor cells on glioma invasion: Profiles of invasive tumor cells after surgery followed by the combination therapy oHSVs+BTZ as a function of the random motility of glioma cells ($D_{1}$) and spreading speed of OVs and BTZ ($D_{v}$) at the final time.

Finally, we investigated the effect of surgical resection when there is a gap in the surrounding CSPGs. Figure 12 shows different patterns of invasion as a function of the diffusivity of cells, OVs and BTZ in response to resection of the tumor at the center followed by combination therapy. The following combinations of $D_{1}$ and $D_{v}$ were used: $\left(D_{1}/10,10D_{v}\right)$ in Figure 12A, $\left(D_{1}^{*},10D_{v}^{*}\right)$ in Figure 12B, $\left(10D_{1}^{*},10D_{v}^{*}\right)$ in Figure 12C, $\left(D_{1}^{*}/10,D_{v}^{*}\right)$ in Figure 12D, $\left(D_{1}^{*},D_{v}^{*}\right)$ in Figure 12E, $\left(10D_{1}^{*},D_{v}^{*}\right)$ in Figure 12F, $\left(D_{1}^{*}/10,D_{v}^{*}/10\right)$ in Figure 12G, $\left(D_{1}^{*},D_{v}^{*}/10\right)$ in Figure 12H, $\left(10D_{1}^{*},D_{v}^{*}/10\right)$ in Figure 12I. Here, $(D_{1}^{*}$ and $D_{v}^{*})$ are the reference diffusion coefficients. The model predicts that different TME conditions such as tissue composition—which affect $D_{1}$ and $D_{v}$—may induce different spatial invasion patterns. When both $D_{1}$ and $D_{v}$ are small, the dynamics are largely controlled by localized infection of OVs and slow migration of tumor cells, leading to limited invasion and tumor cell killing. As $D_{1}$ is increased (from the left panel to the right panel), the increasing diffusion rate of uninfected cells leads to highly invasive phenotypes through the open gap in Q1 (Figure 12G → Figure 12H → Figure 12I). On the other hand, as $D_{v}$ is increased, the dynamics lead to quick spread of OVs in the area, killing more tumor cells (Figure 12G → Figure 12D → Figure 12A). Interestingly, high values of $D_{1}$ and $D_{v}$ lead to the effective eradication of tumor cells in the enclosed area but effective tumor invasion through the gap in Q1, thereby increasing the potential for tumor recurrence at other sites even after surgery and combination therapy (Figure 12C). This type of glioma recurrence in other parts of brain after surgery and OV therapy has been observed in clinical trials by Market et al. [72].

## 4. Discussion

Cellular apoptosis is a typical target of a significant number of anti-cancer chemotherapeutic drugs such as doxorubicin, cisplatin and BTZ. However, patients often develop resistance to such drugs, leading to reduced clinical outcomes. Interestingly, HSV-1 can hijack the usual cellular pathways of ER stress and apoptosis in response to BTZ to override this response [94,95] and induce necroptotic cell death independently of both autophagic cell death and/or apoptosis [21]. While apoptotic cell death involves an immunologically silent death [96,97], necrotic cells release cytokines, resulting in a robust inflammatory response and long term immune response [98,99,100,101]. Interestingly, necroptotic cell death is in the downstream pathway of virus replication and does not affect oHSV replication in vitro [21] but the associated inflammation can also induce a mechanism for pathogen clearance [102]. The combination treatment can induce secretion of several cytokines such as IL1a, in vitro and in vivo [21], which may lead to a significant and long-term anti-tumor immune response. BTZ may also sensitize tumor cells to NK cell- and/or TNF-related apoptosis-inducing ligand (TRAIL)-mediated killing using death receptors, such as DR5 [103,104]. While NK cell treatment in addition to OV injection increases the anti-tumor efficacy, depletion of endogenous NK cells was also shown to enhance the anti-tumor efficacy in the OVs+BTZ combination therapy [51]. Other immune cells such as tumor-associated macrophages (TAMs) [105,106] also play a significant role in regulation of tumor growth and invasion by exchanging signaling molecules such as transforming growth factor-β (TGF-β) and colony stimulating factor 1 (CSF-1), adding complexities in optimizing the OV therapy [107]. Since OV therapy may be able to skew the balance between M2 and M1 activation of TAMs toward the M1 phenotype, thereby activating the anti-tumor immune responses, it may be worthwhile trying to manipulate OVs so as to ‘fully educate’ the existing immune cells [107]. In recent in vitro experiments, TAMs were reported to be able to increase the oncolysis of attenuated measles and mumps virus [108], opening these possibilities. These results illustrate the nonlinear complex aspects of the innate immune system in the TME in regard to regulation of OV therapy [107].

Despite the localized synergistic effects of combination therapies on cancer cell killing in glioma [20,21], its anti-tumor efficacy is not high in the TME, where the heterogenous distribution of extracellular matrix components such as CSPGs [43,47,109], and other structures such as white matter, as well as the host immune system, [51,105,110] play critical roles in the regulation of cell movement [83]. CSPGs, one of the major components of brain ECM, has been shown to inhibit glioma invasion by forming strong tumor-ECM adhesion [44,45,46] and a band of surrounding astrocytes repelled by the dense CSPGs in the tumor center [92]. In the context of OV therapy, CSPGs were shown to inhibit OV spread within the growing glioma [43] and Chase-ABC, a degrading enzyme, was suggested to improve OV spread throughout the tumor core [43,47,111]. Our work shows that a heterogeneous spatial distribution of CSPGs in the brain can affect the anti-tumor efficacy, in particular on invasive glioma cells. Localization of invasive glioma cells to the periphery of the resection site by injecting chemoattractants on the tumor site could improve anti-cancer therapy, as suggested in [92,112,113]. In this regard, recent advances in obtaining vector fields of GBM cell movements may also provide a useful guide for future modeling of tumour growth prior to surgical treatment [114].

In this study we focused on developing BTZ-dependent therapeutic strategies for glioblastoma in the context of OV therapies, but our mathematical model can be applied to other types of cancers. For instance, therapeutic effects of BTZ were studied in colon [115,116], prostate [117,118], breast [119,120], lung [121,122], melanoma [123,124], ovarian [20,125,126], myeloma [25,127,128], head and neck [20] cancers, EBV-associated lymphomas [129], hepatocellular carcinoma [130], and glioma [20,21]. Various types of OVs have been studied in combination with BTZ, including reovirus [131], HSV-1 [20,21,132], adenovirus [133] and VSV [134]. For instance, HSV-1 and reovirus have shown synergistic effects [135] in breast cancer [136] and pancreatic cancer [131]. A combination BTZ+adenovirus treatment led to caspase-dependent apoptosis and suppression of the antiviral immune responses in a hepatocellular carcinoma model [133]. Interestingly, the combination BTZ+OV treatment was found to inhibit VSV replication, showing less than additive cell killing rates in vitro, but additive anti-tumor activity in vivo in myeloma cells [134]. Recently, Yoo et al. [20,21] observed that the combination BTZ+OV therapy can induce the synergistic anti-tumor effects in ovarian and head and neck cancers, as well as in glioma cells and malignant peripheral nerve sheath tumor cells. Other groups have also shown that BTZ therapy can sensitize multiple myeloma therapies to ReoV infection by up-regulating the expression of the viral receptor JAM-A [137,138,139]. Therefore, our mathematical model can provide a framework of investigating the anti-tumor efficacy and developing therapeutic strategies of these cancers by taking into account the organ-specific tumor microenvironmental factors.

In our modeling framework, we can further investigate the effect of the combination BTZ+OV therapy on tumor growth and develop anti-invasion strategies in a complex tumor microenvironment. For instance, the model can be used to predict the anti-tumor responses when some of components in the BTZ-affected signaling network are either promoted or inhibited by anti-cancer drugs, or when tumor ECM components such as CSPGs are modified or manipulated in the brain. Since the location of OV administration is a major determinant of the fundamental characteristics of initial host responses against these armed OVs [140], our mathematical model can be adapted to optimize the injection location of OVs and BTZ in a given tumor location in order to maximize synergistic anti-tumor effects and minimize the negative anti-viral effects from the host defense system. The mathematical model can be also used to optimize the schedule and amount of BTZ and OVs, maximizing the anti-tumor effect and minimizing the administrative costs in the clinic [141,142,143]. However, there exist several disadvantages of the current mathematical approach. For example, the intracellular signaling networks and their regulation in cancerous cells are treated at a cell population level, not at the individual cell level. Therefore, it is quite challenging to localize and control the core IκB-NFκB-Bcl2-Bax-RIP1 signaling network at the individual cell level, especially for highly invasive cells in the tumor microenvironment. In addition, the heterogeneity in the cancer cell population plays an important role in cell motility, cellular invasion, resistance to drugs, and recurrence of the tumor cells. However, it is difficult to take such heterogeneity into account in the current modeling framework. A multi-scale hybrid modeling framework can better represent the cellular process at the individual cell level. For instance, in this work, we did not take into account the detailed biochemical reactions of tumor-ECM adhesion processes. More detailed [CSPG]-[CSGAG] receptor binding kinetics at an individual glioma cell site were modeled in [92]. A useful anti-invasion strategy may emerge by using a hybrid mathematical model [112,113,144,145,146,147,148,149] where important cellular features of highly invasive glioma cells such as cell-ECM adhesion, signaling networks [141,150,151,152,153], cell movement in a dense tissue [154], and interaction with other cells in the TME [105,155,156] can be taken into account. A review of eukaryotic cell motility detailing some of the modeling difficulties appears in [157]. Finally, one of the major components of the core control system in our model, the RIP1-RIP3 axis, is not always intact in all glioblastoma cells. Therefore, the current model may not be applicable in such cases, and we plan to modify the model in order to take into account alternative RIP1-independent cell death mechanisms in a future mathematical model.

Even though BTZ, like other agents, has a low blood-brain barrier (BBB) permeability for treating neurological disorders and spinal muscular atrophy [158,159,160,161,162], recent advances in the delivery method of BTZ improved anti-tumor efficacy by bypassing the BBB, making BTZ a more affordable, effective treatment for GBM [163]. Pre-treatment with an ATP-binding cassette (ABC) transporter inhibitor may also overcome the low biodistribution of BTZs in the CNS by preventing BTZ’s efflux at the BBB [159]. Efficient use of BTZ and combination therapy at different locations in the brain for multi-focal glioma could also improve overall eradication of cancerous cells. For instance, BTZ alone may be strategically applied to kill tumor cells via ER stress in a location with limited access to OVs and the combination therapy OVs+BTZ can be adapted for massive cell killing in more accessible locations, such as the core of a growing tumor.

As it was clearly shown by recent Nobel prize awards, immune therapy and normalization of the immune system greatly enhanced understanding of the role of the immune system as well as the potential of immune therapy. However, recent work by Kim et al. [51] illustrated the dual role of immune systems in the regulation of tumor dynamics in response to a triple combination therapy—OVs+BTZ+NK cells. Furthermore, many immune factors can play a role in regulating killing, replication, and spread of OVs. For instance, IFNα/β can limit virus replication [42,140,164,165,166]. In addition, innate immune cells such as NK cells and macrophages can block spread of OVs throughout the tumor [51,140]. Intravenous, intra-aterial, or intratumoral administration of various types of OVs (vaccinia virus [167], Newcastle disease virus [168], measles virus [169], and adenovirus [170,171]) can be eliminated by the circulating humoral defense agents [172,173]. Intracellular defense mechanism can also limit OV replication [174,175,176,177,178,179,180]. These factors would certainly influence how OV dynamics and anti-tumor effects of therapeutic drugs in the TME are modeled, and some of these will be taken into account in future work. A better understanding of immune-tumor interactions in the TME would certainly lead to development of new therapeutic strategies in OV therapy.

Recurrence of tumors after surgical resection is a major contributing factor to the low survival rate of GBM and conventional treatments such as chemotherapy and radiotherapy are not effective in treating infiltrating glioma cells because of changes in characteristics in signaling pathways such as the apoptotic programs, and in drug resistance [93]. A new innovative strategy in addition to development of OVs, effectively targeting invasive glioma cells, is needed to eradicate cancerous cells and prevent recurrence.

## Figures and Tables

**Table 1:** Model parameters for the intracellular dynamics.

Par	Description	Parameter Values	Ref
$k_{SB}$	Bortezomib signaling scaling factor	1.0 $\times 10^{-1}\;h^{-1}\mu M$	Estimated
$k_{12}$	Inhibition strength of bortezomib by oHSV	$=B^{*}=2.6\times 10^{-2}\;\mu M$	Estimated
$k_{13}$	Inhibition strength of bortezomib by oHSV	7.8 $\times \;10^{-1}$ μM	Estimated
$c_{1}$	Signaling strength of the NFκB-Bcl2 complex	3.64 $\times \;10^{-2}$ $h^{-1}\mu M$	Estimated
$c_{2}$	Signaling strength of Bax	3.43 $\times \;10^{-4}$ $h^{-1}\mu M$	Estimated
$k_{10}$	Signaling strength of RIP1	5.2 $\times \;10^{-1}$ $h^{-1}\mu M$	Estimated
$k_{11}$	Activation rate of RIP1 in the presence of OVs	1.35 $h^{-1}$	Estimated
$k_{1}$	Autocatalytic enhancement rate of IκB	2.08 $\times \;10^{-1}$ $h^{-1}\mu M$	Estimated
$k_{3}$	Autocatalytic enhancement rate of the NFκB-Bcl2 complex	6.91 $\times \;10^{-1}$ $h^{-1}\mu M$	Estimated
$k_{7}$	Autocatalytic enhancement rate of Bax	1.155 $\times \;10^{-2}$ $h^{-1}\mu M$	Estimated
$k_{5}$	Inhibition strength of IκB by the NFκB-Bcl2 complex	8.8 ${\left(\mu M\right)}^{-2}$	Estimated
$k_{6}$	Inhibition strength of the NFκB-Bcl2 complex by IκB	400 ${\left(\mu M\right)}^{-2}$	Estimated
$k_{9}$	Inhibition strength of Bax by the NFκB-Bcl2 complex	4.0 ${\left(\mu M\right)}^{-2}$	Estimated
$k_{2}$	Hill-type parameter	1.0	Estimated
$k_{4}$	Hill-type parameter	1.0	Estimated
$k_{8}$	Hill-type parameter	1.0	Estimated
$\mu_{s}$	Decay rate of IκB	1.0397 $h^{-1}$	[53,54,55]
$\mu_{f}$	Decay rate of NFκB-Bcl2 complex	3.151 $\times \;10^{-1}$ $h^{-1}$	[53,54]
$\mu_{a}$	Decay rate of Bax	2.17 $\times \;10^{-2}$ $h^{-1}$	[53,54,56]
$\mu_{r}$	Decay rate of RIP1	1.444 $\times \;10^{-1}$ $h^{-1}$	[53,54,57]
*k*	Hill type parameter of oHSV switching	0.01 $v^{*}$	Estimated

**Table 2:** Model parameters for the distributed variables. ${}^{‡}$ Dimensionless values.

Par	Description	Parameter Value	Ref.
Diffusion coefficients/Random motility (mm${}^{2}$/h)			
$D_{1}$	random motility of uninfected glioma cells	3.6 $\times 10^{-6}$	[51]
$D_{2}$	Random motility of infected glioma cells	3.6 $\times 10^{-9}$	Estimated
$D_{v}$	random motility of virus (PBS)	3.89 $\times 10^{-2}$	[51,60]
$D_{B}$	diffusion coefficient of bortezomib	2.5 $\times 10^{-2}$	[51]
Production/remodeling rates			
λ	proliferation rate of tumor cells	$4.2\times 10^{-1}$ 1/h	[58], Estimated
$x_{0}$	Carrying capacity of uninfected tumor cells	$=x^{*}$	[47,51,58]
β	Virus infection rate	$2.43\times 10^{-11}$ mm${}^{3}$/(h·virus)	[51,58]
*b*	Burst size of infected cells	**${}^{‡}$** 1.1364 $\times 10^{1}$	[58], Estimated
$\alpha_{1}$	bortezomib-induced viral replication rate	$10^{11}\;{\mathrm{mm}}^{3}/g$	[51]
$I_{B}$	bortezomib supply rate	1.8 $\times 10^{-12}\;{g/(\mathrm{mm}}^{3}\cdot h)$	[51]
Inhibition/degradation/decay rates			
$\beta_{1}$	Bortezomib-induced apoptosis rate of tumor cells	8.0 $\times 10^{8}$ mm${}^{3}$/(g·h)	Estimated
$\beta_{3}$	Necroptosis rate of tumor cells	$1.37\times 10^{3}$ mm${}^{3}$/(g·h)	Estimated
δ	infected cell lysis rate	$8.2\times 10^{-2}\;h^{-1}$	[51,58]
μ	Removal rate of dead cells	1.04 $\times 10^{-1}\;h^{-1}$	[51,58], Estimated
γ	clearance rate of viruses	$1.8\times 10^{-3}\;h^{-1}$	[51,58]
$\mu_{1}$	consumption rate of bortezomib by uninfected tumor cells	2.075$\times 10^{-9}\;h^{-1}$	[51]
$\mu_{2}$	consumption rate of bortezomib by infected tumor cells	2.075$\times 10^{-9}\;h^{-1}$	[51]
$k_{B}$	Hill-type parameter	$=B^{*}$	[51]
$\mu_{B}$	decay rate of bortezomib	$3.47\times 10^{-2}\;h^{-1}$	[61,62]
Reference values of main variables			
$S^{*}$	IκB concentration	0.05 μM	[63,64]
$F^{*}$	Concentration of the NFκB-Bcl2 complex	0.5 μM	[63,64,65,66]
$A^{*}$	Bax concentration	0.1 μM	[67]
$R^{*}$	RIP1 concentration	5.0 μM	[68]
$x^{*}$	Uninfected cell density	$10^{6}\;{\mathrm{cells}/\mathrm{mm}}^{3}$	[51,58,69]
$y^{*}$	Infected cell density	$=x^{*}$	[51,58,69]
$n^{*}$	Dead cell density	$=x^{*}$	[51,58,69]
$v^{*}$	Virus concentration	$2.2\times 10^{8}\;{\mathrm{virus}/\mathrm{mm}}^{3}$	[51,58,69]
$B^{*}$	Bortezomib concentration	$1.0\times 10^{-11}\;{g/\mathrm{mm}}^{3}$	[20,21,51]

## Supplementary Materials

The following are available online at https://www.mdpi.com/2072-6694/11/2/215/s1, Figure S1: (A) A simplied model of the network of IκB-NFκB-Bcl2-Bax-RIP system for anti-apoptosis, apoptosis, and necroptosis of glioma cells [1–5]. (B) Schematic components of IκB, proteasome-NFκB -Bcl2 complex, BAX, and RIP1 are represented by ‘S’, ‘F’, ‘A’, and ‘R’, respectively. Table S1: Reference value used in the model.
