# Supplementary material for: Synergistic Effects of Bortezomib-OV Therapy and Anti-Invasive Strategies in Glioblastoma: A Mathematical Model
Source: Cancers (Basel). 2019 Feb 13;11(2):215. doi: 10.3390/cancers11020215 (PMC6406513; doi:10.3390/cancers11020215)
Supplement: Supplementary file 1 [file cancers-11-00215-s001.pdf]

Supplementary Material

Mathematical Model Development and Analysis

Yangjin Kim, Junho Lee, Donggu Lee, and Hans Othmer

1 Nondimensionalization

1.1 Intracellular model (S, F, A, R)

Using a mathematical model abstracted from the complex network in Figure S1, we illustrate the biochemical mechanisms and conditions under which these signaling molecules operate. A system of ordinary differential equations was utilized to understand the intracellular dynamics. In order to incorporate the signaling network we simplified the network as shown in Figure S1A as follows: We merged all the regulatory network between proteasome/Bcl2 and NFκB -Bcl2 into one component (NFκB-Bcl2 complex; middle green box with dashed line in Figure S1A) while we kept IκB (dashed yellow box in Figure S1A) and Bax (dashed blue box in Figure S1A) in separate modules. The corresponding mathematical model network is shown in Figure S1B. We refer to the interactions represented by edges in Figure S1B as the core control system. By convention, the kinetic interpretation of arrows and hammerheads in the chemical network signify induction (arrow) and inhibition (hammerhead).

Let the variables $S(t), F(t), A(t)$ and $R(t)$ be activities of IκB, NFκB-Bcl2 complex, Bax, and RIP1, respectively, at time t. The scheme includes autocatalytic activities of IκB (S), NFκB-Bcl2 complex (F), Bax (A), and RIP1 (R), protein degradation of those key molecules, mutual inhibition between IκB and NFκB-Bcl2 complex and inhibition of Bax activity by NFκB-Bcl2 complex and RIP1, and activation of RIP1 by NFκB-Bcl2 complex in the presence of OVs and bortezomib. Based on biological observations, we write the phenomenological equations for the rate change of those key modules (S, F, A, R) as follows:

$$\frac{dS}{dt} = k_{SB}\frac{B}{k_{12} + k_{13}[oHSV]} + \frac{k_1 k_2^2}{k_2^2 + k_5 F^2} - \mu_s S, \tag{1}$$

$$\frac{dF}{dt} = c_1 + \frac{k_3 k_4^2}{k_4^2 + k_6 S^2} - \mu_f F, \tag{2}$$

$$\frac{dA}{dt} = c_2 + \frac{k_7 k_8^2}{k_8^2 + k_9 F^2} - \mu_a A. \tag{3}$$

$$\frac{dR}{dt} = k_{10} + k_{11}[oHSV]F - \mu_r R. \tag{4}$$

where the first term in Eq. (1) represents the signaling pathways from bortezomib to IκB in the absence and presence of OVs, k_{SB} is the signaling strength of bortezomib, B is the bortezomib level, k_{12} is a scaling factor for inhibition of the bortezomib signaling, k_{13} is the inhibition strength of bortezomib signaling by OVs, [oHSV] is a biochemical switch for oncolytic viruses with [oHSV] $= \frac{v}{k+v}$ where v is the OV density, as introduced below, and k is the Hill type parameter, giving [oHSV] $= 0$ (1) in the absence (presence) of virus, c_1, c_2, k_{10} are the signaling pathways to the proteasome-NFκB -Bcl-2 complex, Bax, and RIP1, respectively, k_1, k_2, k_3 are the autocatalytic enhancement parameters for IκB, proteasome-NFκB-Bcl-2 complex and Bax, respectively, k_2, k_4, k_8 are the Hill-type inhibition saturation parameters from the counter part of IκB, proteasome-NFκB -Bcl-2 complex and Bax, respectively, k_5 is the inhibition strength of IκB by the proteasome-NFκB-Bcl-2 complex, k_6 is the inhibition strength of the proteasome-NFκB-Bcl-2 complex by IκB, k_9 is the inhibition strength of the Bax by the , and finally, $\mu_s, \mu_f, \mu_a, \mu_r$ are decay rates of IκB, proteasome-NFκB-Bcl-2 complex, Bax, RIP1, respectively.

By performing the following non-dimensionalization:

$$\bar{t} = \mu_s t, \bar{S} = \frac{S}{S^*}, \bar{F} = \frac{F}{F^*}, \bar{A} = \frac{A}{A^*}, \bar{R} = \frac{R}{R^*}, \lambda_B = \frac{k_{SB}B^*}{S^*\mu_s k_{12}},\ \alpha = \frac{k_{13}}{k_{12}},\ \sigma_1 = \frac{k_1}{S^*\mu_s},\ \sigma_9 = k_2,$$
$$\sigma_4 = k_5(F^*)^2, \sigma_7 = \frac{c_1}{F^*\mu_s},\ \sigma_2 = \frac{k_3}{F^*\mu_s},\ \sigma_{10} = k_4,\ \sigma_5 = k_6(S^*)^2,\ \omega_1 = \frac{\mu_f}{\mu_s},\ \sigma_8 = \frac{c_2}{A^*\mu_s}, \tag{5}$$
$$\sigma_3 = \frac{k_7}{A^*\mu_s},\ \sigma_{11} = k_8,\ \sigma_6 = k_9(F^*)^2,\ \omega_2 = \frac{\mu_a}{\mu_s},\ \sigma_{12} = \frac{k_{10}}{R^*\mu_s},\ \sigma_{13} = \frac{k_{11}F^*}{\mu_s R^*},\ \omega_3 = \frac{\mu_r}{\mu_s},\ \bar{k} = \frac{k}{v^*},$$

of Eqs. (1)-(4), we obtain the dimensionless equations for IκB ($\bar{S}$), NFκB-Bcl2 ($\bar{F}$), Bax ($\bar{A}$), RIP1 ($\bar{R}$) with a set of essential control parameters:

$$\frac{d\bar{S}}{d\bar{t}} = \lambda_B \frac{B}{1+\alpha[oHSV]} + \frac{\sigma_1\sigma_9^2}{\sigma_9^2+\sigma_4\bar{F}^2} - \bar{S}, \tag{6}$$
$$\frac{d\bar{F}}{d\bar{t}} = \sigma_7 + \frac{\sigma_2\sigma_{10}^2}{\sigma_{10}^2+\sigma_5\bar{S}^2} - \omega_1\bar{F}, \tag{7}$$
$$\frac{d\bar{A}}{d\bar{t}} = \sigma_8 + \frac{\sigma_3\sigma_{11}^2}{\sigma_{11}^2+\sigma_6\bar{F}^2} - \omega_2\bar{A}, \tag{8}$$
$$\frac{d\bar{R}}{d\bar{t}} = \sigma_{12} + \sigma_{13}[oHSV]\bar{F} - \omega_3\bar{R}. \tag{9}$$

where [oHSV] $= \frac{\bar{v}}{\bar{k}+\bar{v}}$ where $\bar{v}$ is the dimensionless OV density, as introduced below.

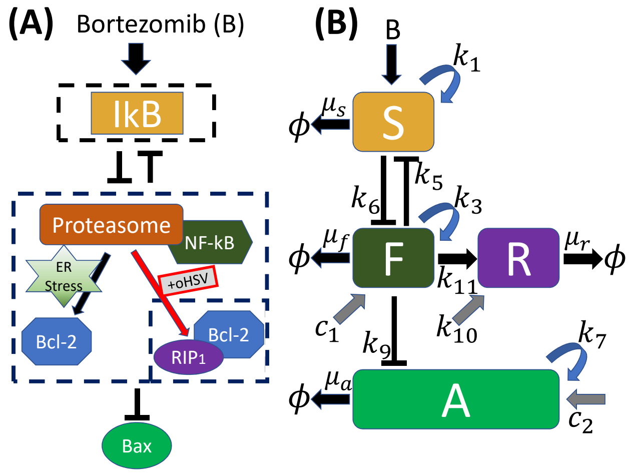

Figure S1. (A) A simplified model of the network of IκB-NFκB-Bcl2-Bax-RIP system for anti-apoptosis, apoptosis, and necroptosis of glioma cells [1–5]. (B) Schematic components of IκB, proteasome-NFκB -Bcl2 complex, BAX, and RIP1 are represented by 'S', 'F', 'A', and 'R', respectively.

Var	Description	Dimensional value	Ref
L	Length scale	3 mm	[1,2,5]
T	Time scale	1.0 h	[5]
D	Characteristic diffusion coefficient	$1.5 \times 10^{-5} cm^2/s$	[5]
x^*	Uninfected cell density	10^6 $cells/mm^3$	[5–7]
y^*	Infected cell density	$= x^*$	[5–7]
n^*	Dead cell density	$= x^*$	[5–7]
v^*	Virus concentration	2.2×10^8 $virus/mm^3$	[5–7]
B^*	Bortezomib concentration	1.0×10^{-11} g/mm^3	[1,2,5]
S^*	IκB concentration	0.05 μM	[8,9]
F^*	concentration of the NFκB-Bcl2 complex	0.5 μM	[8–11]
A^*	Bax concentration	0.1 μM	[12]
R^*	RIP concentration	5.0 μM	[13]

Table S1. Reference value used in the model.

1.2 Diffusible variables (x, y, n, v, B)

The following nondimensionalization was used for the governing equations (8)-(12) in the main text

$$\begin{aligned}
&\bar{t} = \frac{t}{T},\ T = \frac{1}{\mu_s}, \Theta = x^*,\ \bar{x} = \frac{x}{\Theta},\ \bar{y} = \frac{y}{\Theta},\ \bar{n} = \frac{n}{\Theta},\ \bar{v} = \frac{v}{v^*},\ \bar{D}_1 = \frac{D_1}{\tilde{D}},\ \bar{D}_2 = \frac{D_2}{\tilde{D}},\ \bar{D}_v = \frac{D_v}{\tilde{D}},\\
&\bar{D}_B = \frac{D_B}{\tilde{D}},\ \bar{\lambda} = T\lambda,\ \bar{x}_0 = \frac{x_0}{x^*},\ \bar{\beta} = Tv^*\beta,\ \bar{\beta}^\dagger = \frac{Tx^*v^*\beta}{y^*},\ \bar{\beta}_1 = TB^*\beta_1,\ \bar{\beta}_3 = Tv^*\beta_3,\\
&\bar{\beta_3}^\dagger = \frac{Tx^*v^*\beta_3}{y^*},\ \bar{\delta} = T\delta,\ \bar{\delta}^\dagger = \frac{T\delta y^*}{n^*},\ \bar{\mu} = T\mu,\ \bar{b} = \frac{by^*}{v^*},\ \bar{\alpha}_1 = B^*\alpha_1,\ \bar{\gamma} = T\gamma,\\
&\bar{I}_B = \frac{TI_B}{B^*},\ \bar{\mu}_1 = \frac{Tx^*\mu_1}{B^*},\ \bar{\mu}_2 = \frac{Ty^*\mu_2}{B^*},\ \bar{k}_B = \frac{k_B}{B^*},\ \bar{\mu}_B = T\mu_B,
\end{aligned} \tag{10}$$

to get the dimensionless model equations:

$$\frac{\partial \bar{x}}{\partial \bar{t}} = \bar{\nabla} \cdot (\bar{D}_1 \bar{\nabla} \bar{x}) + \bar{\lambda}\bar{x}(1 - \bar{x}/\bar{x}_0) - \bar{\beta}\bar{x}\bar{v} - \bar{\beta}_1 \bar{B}\bar{x} I_{apop} - \bar{\beta}_3 \bar{x}\bar{v} I_{Necrop} \tag{11}$$

$$\frac{\partial \bar{y}}{\partial \bar{t}} = \bar{\nabla} \cdot (\bar{D}_2 \bar{\nabla} \bar{y}) + \bar{\beta}^\dagger \bar{x}\bar{v} - \bar{\delta}\bar{y} + \bar{\beta}_3^\dagger \bar{x}\bar{v} I_{Necrop} \tag{12}$$

$$\frac{\partial \bar{n}}{\partial \bar{t}} = \bar{\delta}^\dagger \bar{y} - \bar{\mu}\bar{n}, \tag{13}$$

$$\frac{\partial \bar{v}}{\partial \bar{t}} = \bar{\nabla} \cdot (\bar{D}_v \bar{\nabla} \bar{v}) + \bar{b}\bar{\delta}\bar{y}(1 + \bar{\alpha}_1 \bar{B}) - \bar{\gamma}\bar{v}, \tag{14}$$

$$\frac{\partial \bar{B}}{\partial \bar{t}} = \bar{\nabla} \cdot (\bar{D}_B \bar{\nabla} \bar{B}) + \bar{I}_B - (\bar{\mu}_1 \bar{x} + \bar{\mu}_2 \bar{y}) \frac{\bar{B}}{\bar{k}_B + \bar{B}} - \bar{\mu}_B \bar{B}, \tag{15}$$

*Note that $\beta^\dagger = \beta, \beta_3^\dagger = \beta_3, \delta^\dagger = \delta$ under the assumption of $x^* = y^* = n^*$; otherwise $\beta^\dagger, \beta_3^\dagger, \delta^\dagger$ are different from β, β_3, δ, respectively.
Table S1 lists the reference values in the model.

Boundary Condition:

We take no-flux boundary condition for all diffusible variables (x, y, v, B) on the boundary $\Gamma = \partial\Omega$

$$(D_1 \nabla x) \cdot \nu = 0, \quad (D_2 \nabla y) \cdot \nu = 0, \quad (D_v \nabla v) \cdot \nu = 0, \quad (D_B \nabla B) \cdot \nu = 0, \tag{16}$$

where ν is the outer normal vector.

2 Parameter estimation

$\omega_1, \omega_2, \omega_3$: Half-life of suppressor (IκB) is in the range of (10-40) *mins* [14–16]. By taking 40 *mins* of half-life, we get $\mu_s = ln(2)/(40\ min) = 1.0\ h^{-1}$. We take 2.2 *hours* of half-life for the NFκB-Bcl2 complex, leading to $\mu_f = 0.315\ h^{-1}$ and $\omega_1 = \frac{\mu_f}{\mu_s} = 0.3$. Half-life of Bax is approximated to be in the range of (6-32) hours [17], leading to $\mu_a = ln(2)/(32\ h) - ln(2)/(6\ h) = (0.0217 - 0.1155)\ h^{-1}$. We take $\mu_a = 0.02\ h^{-1}$, resulting in $\omega_2 = \frac{\mu_a}{\mu_s} = 0.02$. Half-life of RIP1 was estimated to be (1.2-5) hours [18]. By taking 4.8 *hour* of half life, we have $\mu_r = 0.1444\ h^{-1}$ and $\omega_3 = \frac{\mu_r}{\mu_s} = 0.139$

D_1, D_2, D_v: Diffusion coefficient of tumor cells D_1 is $3.6 \times 10^{-6}\ mm^2/h$ [5]. Random motility of infected cells is very limited due to OV infection and we take much slower value, $D_2 = D_1/1000$. From the approximation $D_v \sim 10^4 D$ [19], we get $D_v = 3.89 \times 10^{-2}\ mm^2/h$.

α_1: We assume that effect of OV replication doubles when $B = B^*$, (*i.e.,* , $1 + \alpha_1 B^* = 2$), based on experimental observation [2]. So, we take $\alpha_1 = 10^{11}\ mm^3/g$.

k_B: We take the bortezomib level (B^*) to indicate the instant consumption of bortezomib by tumor cells, leading to $k_B = B^* = 1.0 \times 10^{-11}\ g/mm^3$.

μ_B: The average half-life of Bortezomib was estimated to be 10-31 hours [20]. The mean removal half-life of after the first dose was in the range of 9-15 hours at the dose level 1.45-2.00 mg/m^2 in cancer patients [21]. We take 20 h, leading to $\mu_B = \frac{ln2}{20\ h} = 3.47 \times 10^{-2}/h$.

μ_1, μ_2: While proteasome inhibitor, bortezomib (B), has a relatively long half-life of 31 hours, internalization of this key molecule into an infected tumor cell and infection of tumor cells happen at a faster time scale. Therefore, we assume that the consumption rate in the bortezomib equation is much higher than natural decay term, resulting in $(\mu_1 x + \mu_2 y)\frac{1}{2} > \mu_B B$ with an approximation of $\frac{B}{k_1+B} \sim 0.5$. By setting $\mu_1 = \mu_2$ and an estimation of $x + y \sim 0.8 \times 10^{-3}\ g/mm^3$, we get $\mu_1 = \mu_2 = \frac{0.166 \times 10^{-8}}{0.8} \frac{1}{h} = 2.075 \times 10^{-9}/h$.

References

1. Yoo JY, Hurwitz BS, Bolyard C, Yu J, Zhang J, Selvendiran K, et al. Bortezomib-Induced Unfolded Protein Response Increases Oncolytic HSV-1 Replication Resulting in Synergistic Antitumor Effects. Clin Cancer Res. 2014;20(14):3787–3798.

2. Yoo JY, Jaime-Ramirez AC, Bolyard C, Dai H, Nallanagulagari T, Wojton J, et al. Bortezomib treatment sensitizes oncolytic HSV-1 treated tumors to NK cell immunotherapy. Clin Cancer Res. 2016;pii: clincanres.:1003.2016.

3. Ashkenazi A, Salvesen G. Regulated cell death: signaling and mechanisms. Annu Rev Cell Dev Biol. 2014;30:337–356.

4. Najafov A, Chen H, Yuan J. Necroptosis and Cancer. Trends Cancer. 2017;3(4):294–301.

5. Kim Y, Yoo JY, Lee TJ, Liu J, Yu J, Caligiuri MA, et al. Complex role of NK cells in regulation of oncolytic virus-bortezomib therapy. Proc Natl Acad Sci USA. 2018;115(19):4927–4932.

6. Friedman A, Tian JP, Fulci G, Chiocca EA, Wang J. Glioma virotherapy: effects of innate immune suppression and increased viral replication capacity. Cancer Res. 2006;66(4):2314–9.

7. ODonoghue JA, Bardies M, Wheldon TE. Relationships between tumor size and curability for uniformly targeted therapy with beta-emitting radionuclides. J Nucl Med. 1995;36(10):1902–9.

8. Lipniacki T, Paszek P, Brasier AR, Luxon B, Kimmel M. Mathematical model of NF-kappaB regulatory module. J Theor Biol. 2004;228(2):195–215.

9. Lee EG, Boone DL, Chai S, Libby SL, Chien M, Lodolce JP, et al. Failure to regulate TNF-induced NF-kappaB and cell death responses in A20-deficient mice. Science. 2000;289(5488):2350–4.

10. Mothes J, Busse D, Kofahl B, Wolf J. Sources of dynamic variability in NF-kB signal transduction: a mechanistic model. Bioessays. 2015;37(4):452–62.

11. Xue X, Xia W, Wenzhong H. A modeled dynamic regulatory network of NF-?B and IL-6 mediated by miRNA. Biosystems. 2013;114(3):214–8.

12. Kirkland RA, Saavedra GM, Cummings BS, Franklin JL. Bax regulates production of superoxide in both apoptotic and nonapoptotic neurons: role of caspases. J Neurosci. 2010;30(48):16114–27.

13. Li J, McQuade T, Siemer AB, Napetschnig J, Moriwaki K, Hsiao YS, et al. The RIP1/RIP3 necrosome forms a functional amyloid signaling complex required for programmed necrosis. Cell. 2012;150(2):339–50.

14. Bergqvist S, Ghosh G, Komives EA. The IkBa/NF-kB complex has two hot spots, one at either end of the interface. Protein Science. 2008;17:2051–2058.

15. Mathes E, O?Dea EL, Hoffmann A, Ghosh G. NF-kB dictates the degradation pathway of IkBa. The EMBO Journal. 2008;27:1357–1367.

16. Krappmann D, Scheidereit C. Regulation of NF-kappa B activity by I kappa B alpha and I kappa B beta stability. Immunobiology. 1997;198(1-3):3–13.

17. Xin M, Deng X. Nicotine Inactivation of the Proapoptotic Function of Bax through Phosphorylation. THE JOURNAL OF BIOLOGICAL CHEMISTRY. 2005;280(11):10781–10789.

18. Wang Q, Chen W, Xu X, Li B, He W, Padilla MT, et al. RIP1 potentiates BPDE-induced transformation in human bronchial epithelial cells through catalase-mediated suppression of excessive reactive oxygen species. Carcinogenesis. 2013;34(9):2119–2128.

19. Jacobsen K, Russell L, Kaur B, Friedman A. Effects of CCN1 and macrophage content on glioma virotherapy: A mathematical model. Bull Math Biol. 2015;77(6):984–1012.

20. Leveque D, Carvalho MCM, MALOISEL F. Clinical Pharmacokinetics of Bortezomib. In vivo. 2007;21:273–278.

21. Kane RC, Bross PF, Farrell AT, Pazdur R. The mean elimination half-life of bortezomib after the first dose ranged from 9-15 hours at doses ranging from 1.45?2.00 mg/m2 in patients with advanced malignancies. The Oncologist. 2003;8:508–513.
